# Nutritional Composition and Phytochemical Evaluation of Some Selected Wild Edible Plants in Tach Gaint District, Northwestern Ethiopia

**DOI:** 10.1155/2023/6670648

**Published:** 2023-10-16

**Authors:** Yalew Yiblet, Endale Adamu

**Affiliations:** ^1^Department of Biology, College of Natural and Computational Sciences, Mekdela Amba University, P.O. Box 32, Tulu Awlia, Ethiopia; ^2^Department of Biology, College of Natural and Computational Sciences, Debre Tabor University, P.O. Box 272, Debre Tabor, Ethiopia

## Abstract

The objective of the study was to evaluate the nutritional, mineral, and phytochemical analyses of some selected wild edible plants from Tach Gaint District, Northwest Ethiopia. Proximate composition parameters (moisture, ash, crude fibre, crude fat, crude protein, carbohydrate, and energy) were evaluated using the methods of the Association of Official Analytical Chemists, and elemental analysis was performed using the atomic absorption spectroscopy technique. Results from the nutritional analysis show that *Erucastrum abyssinicum* leaves had the highest crude protein content (17.47 ± 0.03 g/100 g), followed by *Amaranthus graecizans* (14.97 ± 0.03 g/100 g). The maximum moisture content (40.8 ± 0.00 g/100 g) and ash content (24.70 ± 0.15 g/100 g) were reported in the leaves of *Amaranthus graecizans*. The young shoots of *Rumex abyssinicus* had the highest crude fat content (14.07 ± 0.03 g/100 g) and the highest fibre content (34.70 ± 0.25 g/100 g), while the fruits of *Opuntia ficus-indica* had the highest amount of utilisable carbohydrate (44.4 ± 0.00 g/100 g) and the estimated energy value (326.4 ± 0.00 Kcal/100 g). Calcium was detected in considerable proportions (754.9 ± 0.23 mg/100 g) followed by iron (31.63 ± 0.03 mg/100 g) in *Urtica simensis* leaves and zinc content (3.09 ± 0.02 mg/100 g) in young shoots of *Rumex abyssinicus*. Qualitative phytochemical screening, alkaloids, phenols, flavonoids, triterpenes, saponins, and tannins were found in the methanolic extract of the plants. The results of this study suggest that the consumption of such nutrient-rich wild edible plants could help add a remarkable amount of nutrient and mineral in the human diet.

## 1. Background

In many developing nations, millions of people lack access to enough food to meet their daily needs, and many others are deficient in one or more micronutrients. Plants are crucial to human survival and are the basis of life on Earth [1]. Wild floras are home to a wide array of helpful plants that have long served as a valuable source of needs. The variety of wild plants allows for variety in family diets and helps to ensure the dietary diversity in household food supplies [2]. Some species are marketable and offer the opportunity to increase household income in addition to their nutritional benefits. Throughout the world, the use of such edible species for diet and nutrition is considered an essential and significant aspect of the values and traditions [3].

Consumers may have access to a wider variety of nutritionally beneficial phytoconstituents from locally available wild edible plants. They provide a wide range of phytochemicals, including phenols, tannins, flavonoids, terpenoids, polysaccharides, steroids, saponins, and alkaloids, that can be a source of energy, fibre, and micronutrients [4]. For example, phenolic compounds play a crucial role in the prevention of inflammation, antimicrobial effects, and powerful antioxidant or free radical scavenging activities [5]. Alkaloids are strong medicines that have anti-inflammation, antimalarial, and antimicrobial properties [6]. The antioxidant properties of phenolic compounds are crucial for the healthy operation of human and plant cells [7].

Even in the most developed parts of the world, people regularly use edible wild plants as a supplement to healthy diets, in addition to using them in underdeveloped communities. Approximately one billion people in the world consume wild foods from daily sources. Over 7,000 species have reportedly been used as food in human history, according to ethnobotanical studies on wild edible plants [8]. In many countries, including China, India, Southeast Asian nations, Africa, and Australia, various wild edible plant species are consumed in conjunction with cultivated plant species, despite the fact that these are not commonly accessible [9].

Malnutrition is a problem for public health in many developing nations because it increases the risk of chronic diseases, stunting, and eventually nutritional abnormalities. Based on the accessibility of food, it is also recognized that a global health problem exists due to the lack of minerals such as calcium and zinc [7]. Ethiopia is one of the countries suffering from food insecurity and relies on wild edible plants in addition to cultivated grains to meet nutritional demands, especially in low-income areas. According to Luelkal et al. [10], there are about 413 different kinds of wild food plants consumed in Ethiopia. Research that focuses mostly on the dietary characteristics of wild edible plants still does not receive sufficient attention in Tach Gaint District. The current study evaluates the nutritional value of five wild edible plants, *Rumex abyssinicus*, *Amaranthus graecizans*, *Erucastrum abyssinicum*, *Urtica simensis*, and *Opuntia ficus*-*indica*. The main objective of the study was to determine the biochemical characteristics of these wild edible undomesticated plants and assess their nutritional value, which consisted of protein, carbohydrate, fibre, lipids, mineral, and phytochemical components.

## 2. Materials and Methods

### 2.1. Collection of Plant Materials

The study was conducted at Tach Gaint District. In terms of latitude and longitude, this area is located between 11° 29′ 59.99°–11° 15′ 36° and 38° 14′ 60°–38° 37′ 42°, respectively. Wild edible plants were collected from various locations in the district. During September 2020 to November 2021, the species were carefully recognized in accordance with the appropriate literature on the flora of Ethiopia and Eritrea. Healthy *Amaranthus graecizans* leaves, *Erucastrum abyssinicum* leaves, *Urtica simensis* leaves, *Opuntia ficus-indica* fruits, and *Rumex abyssinicus* young shoots were the five wild edible plant samples collected for nutritional and physicochemical evaluation.

### 2.2. Sample Preparation

The plant parts were carefully cleaned with water before being shade-dried at 25–30°C. Fresh plant parts were obtained for the experiment, cleaned with tap water to eliminate dust, and air-dried for a week. Before being shipped to Mekdela Amba University's Biology Department Laboratory in Ethiopia, each wild edible plant was coded, dried in a shed, crushed, ground to a fine powder in a grinder, and stored in an airtight container.

### 2.3. Nutritional Analysis

#### 2.3.1. Moisture Content

The moisture content of each sample was determined according to the AOAC [11] standards using an oven. A clean and empty crucible and its porcelain lids were dried in a drying oven at 100°C for one hour, cooled in a desiccator for roughly 30 minutes, and weighed. An entire amount of 5.0 g of each sample was weighed in triplicate. The crucible and its contents were put in a drying oven and dried for three hours at 105°C. After drying, the samples were cooled in a desiccator for 30 minutes before being reweighed until a steady weight was achieved. The percentage moisture content was then calculated using the following formula:

(1) $$\begin{array}{c} \text{Moisture }\%=\frac{W3-W1}{W2-W1}\times 100, \end{array}$$

where *W*1 is the weight of the empty crucible, *W*2 is the weight of the sample and crucible, and *W*3 is the weight of the dry sample and crucible.

#### 2.3.2. Ash Contents

Total ash was determined according to the official AOAC method [12, 13]. Two grams of each sample were placed in porcelain crucibles, weighed, and burnt at 550°C for 30 minutes in a muffle furnace. The ashed samples were then taken out, allowed to cool in a desiccator, and then weighed. Equation (2) was used to calculate the percentage of ash:

(2) $$\begin{array}{c} \text{Ash }\%=\frac{W3-W1}{W2-W1}\times 100, \end{array}$$

where *W*1 is the weight of the crucible, *W*2 is the weight of the sample and the crucible, and *W*3 is the weight of the ash and the crucible.

#### 2.3.3. Crude Fat

To determine the fat content, the Soxhlet extraction method was performed. Each plant sample was weighed on the filter paper (Whatman No. 2) and placed in a dry extraction thimble, which was then placed in the Soxhlet extraction tube [14].

(3) $$\begin{array}{c} \text{Fat }\%=\frac{W1-W2}{W}\times 100, \end{array}$$

where *W*_1_ = initial weight of the flask and sample; *W*_2_ = final weight of the flask and sample; *W* = weight of the sample (g).

#### 2.3.4. Crude Fibre

A dried plant sample was mixed with an acetone and ethanol mixture, and then, fibre was determined using AOAC methods [15].

(4) $$\begin{array}{c} \text{Fibre }\%=\frac{W1-W2}{W}\times 100, \end{array}$$

where *W*2 = weight of (crucible + sample) after drying, *W*1 = weight of (crucible + sample) after ashing, and *W* = weight of the sample.

#### 2.3.5. Crude Protein

The amount of crude proteins in the plant samples was determined using the micro-Kjeldahl method [16]. Using the following formula, the percentage of nitrogen was calculated:

(5) $$\begin{array}{c} \text{Protein }\%=\frac{\left(V2-V1\right)\text{MHCl}}{W}\times 14\times 6.25\times 100, \end{array}$$

where *V*2 = volume (ml) of hydrochloric acid solution required for the test sample, *V*1 = volume of hydrochloric acid required for the blank test, MHCl = morality of hydrochloric acid, *W* = weight in grams of the test sample, 6.25 = nitrogen conversion factor of protein, and 14 = atomic mass of nitrogen.

#### 2.3.6. Carbohydrate Content

The carbohydrate content was determined by calculation using the different method: carbohydrate content = (100 − % (moisture + crude protein + crude fat + ash + crude fibre)).

#### 2.3.7. Gross Energy Value

The gross energy values (Kcal/100 g samples) of the wild edible plants were estimated using the factors for protein (4 Kcal/g), fat (9 Kcal/g), and carbohydrate (4 Kcal/g). The equation is gross energy value = (percent crude protein × 4) + (percent fat content × 9) + (percent carbohydrate × 4).

### 2.4. Determination of Minerals

Mineral analyses were carried out using the standard method for eliminating organic matrix in a muffle furnace [17]. The residual ash was dissolved in diluted acid, and the analysed concentration was calculated using an atomic absorption spectrophotometer (PG Instruments Ltd., United Kingdom, and model PG-990) for calcium, zinc, and iron at absorbances of 422.7 nm, 213.9 nm, and 248.3 nm, respectively.

### 2.5. Phytochemical Analysis

Through qualitative examination of some selected plants, a variety of phytochemicals observed in the methanolic extract, including saponins, tannins, phenols, and alkaloids, were investigated. This was performed following the accepted methods [18].

### 2.6. Statistical Analysis

The results of the nutritional and elemental analyses were performed in triplicate, and the values are shown as the mean ± standard deviation. The statistical analysis was performed using the statistical package for the social sciences (SPSS) version 20.

## 3. Results and Discussion

### 3.1. Nutrient Composition of Edible Wild Plants

**Figure 1:**
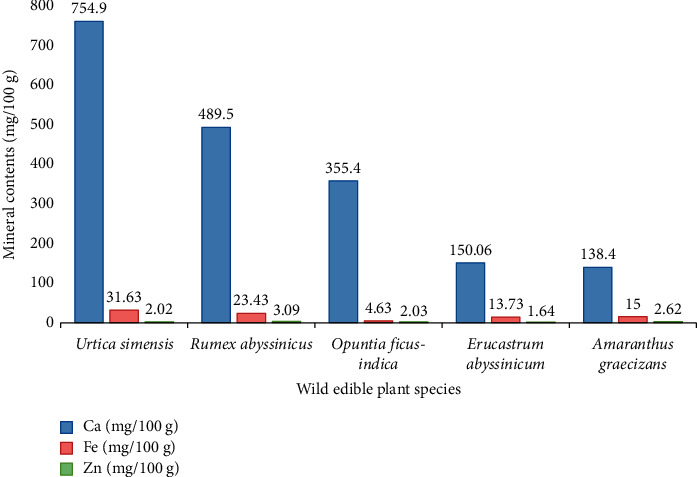
Mineral contents of five wild edible plants in the study area.

When a crop is considered as a food source, nutritional value takes precedence. The estimated dry matter composition of five wild edible plants is reported in Table 1. The study result revealed that the highest estimated energy content was found in the fruits of *Opuntia ficus-indica* (326.4 ± 0.00 Kcal/100 g), followed by the leaves of *Erucastrum abyssinicum* (263.4 ± 0.30 Kcal/100 g). In this finding, the estimated energy was higher than the earlier reported value (217.77 Kcal/100 g) by [19] in *Pyrenacantha klaineana* leaves consumed as food in Northern Angola. The crude protein content in *Erucastrum abyssinicum* (17.47 ± 0.19 g/100 g) is quite similar to that obtained in leaves of *Diplazium esculentum* (17.42 ± 0.19 g/100 g) in other studies [20]. The crude protein content of *Amaranthus graecizans* leaves was (14.97 ± 0.03 g/100 g), which is in agreement with the protein content reported in the same species (28.5 ± 0.2 g/100 g) [21]. The same species may vary depending on a number of variables, such as the amount of nutrients in the soil, the ecological setting, or the stage of growth of the species at the time of collection. The leaves of *Amaranthus graecizans* had the highest ash content (24.7 ± 0.15 g/100 g). All plant species have a wide range of total ash content. A high value of ash indicates a rich supply of minerals in the plant because the ash content is a measure of mineral composition [22]. The fruits of *Opuntia ficus-indica* (44.4 ± 0.00 g/100 g), *Erucastrum abyssinicum* (32.99 ± 0.00 g/100 g), and *Urtica simensis* (26.53 ± 0.12 g/100 g) had the highest carbohydrate content, respectively. This finding contradicts with findings from India [23] that were reported for *Melodinus khasianus* fruits (80.88 ± 0.13%) and *Piper pedicellatum* leaves (63.06 ± 0.06%). The fat content of *Urtica simensis* leaves (4.40 ± 0.6 g/100 g) was comparatively low compared to wild fruits like *Sclerocarya birrea* (9.6 ± 0.17 g/100 g) [24]. A healthy diet should contain 1-2% of its calories from fat, according to various theories. This is because the low crude fat content of the young shoot of *Urtica simensis* shows that it might shield people from chronic diseases [25]. The leaves of *Erucastrum abyssinicum* had the least crude fibre content (7.80 ± 0.2 g/100 g), whereas the crude fibre content of *Rumex abyssinicus* was (34.70 ± 0.25 g/100 g). The amount of crude fibre reported from raw anchote (0.60 g/100 g) [26] is much smaller than the fibre contents of the studied wild edible plants. Dietary fibre lowers the risk of colon cancer and helps humans' good gut flora flourish [27]. *Rumex abyssinicus*, due to its relatively high fibre content, may enhance digestion, promote peristaltic motion, and lessen constipation. Some diseases linked to metabolic abnormalities may be less common as a result of a high-fibre diet [28].  Figure 1 displays the mineral composition of the plant parts that can be eaten. The leaves of *Urtica simensis* were rich in calcium content (754.9 ± 0.23 mg/100 g), followed by the young shoots of *Rumex abyssinicus* (489.5 ± 0.32 g/100 g). With respect to findings from other species reported by Mokria et al. [29], the current finding shows that the accumulation of calcium in *Urtica simensis* is significantly higher. It is also necessary for the regular functioning of the heart muscles, blood coagulation, milk coagulation, and cell permeability modulation [25]. The highest iron content in *Urtica simensis* leaves (31.63 ± 0.03 mg/100 g) was similar to that in *Corchorus olitorius* leaves (31.64 ± 6.24 mg/100 g) reported by [30] in Northern Uganda. When used as a dietary supplement, the amount of iron found in *Urtica simensis* leaves was able to offer almost twice as much as the recommended daily allowance (RDA) of 18 mg/day required by people to make up for a nutritional deficiency in iron [31]. A widespread nutritional issue affecting many people worldwide is iron deficiency. Chronic bleeding, infections, a lack of bioavailable iron, folic acid, vitamin A or vitamin B12, pregnancy, increased nutritional needs during growth periods, and menstrual losses in women of reproductive age are the main causes [32]. The zinc (Zn) concentration of *Erucastrum abyssinicum* leaves (1.64 ± 0.01 mg/100 g) was higher than that of *Rumex abyssinicus* young shoots (3.09 ± 0.02 mg/100 g). The zinc content of the young shoot of *Rumex abyssinicus* is relatively high and can supply around 22% of the daily needs of children and adults because the RDA for zinc is 4–14 mg/day [33]. Several cellular processes, including healthy growth, brain development, behavioural response, bone creation, and wound healing, require trace metal zinc [34].

The qualitative phytochemical analysis of the selected wild edible plants is shown in Table 2. The highest levels of phenols and alkaloids were found in the fruits of *Opuntia ficus*-*indica* and the leaves of *Amaranthus graecizans*, respectively. The level of saponins was the highest in the leaves of *Urtica simensis.* Triterpenes and tannins are not present in *Erucastrum abyssinicum,* whereas the study showed that *Amaranthus graecizans*, *Opuntia ficus-indica, Rumex abyssinicus,* and *Urtica simensis* possess all the tested phytochemicals such as phenols, alkaloids, tannins, triterpenes, saponins, and flavonoids. The results of the qualitative phytochemical analysis of *Amaranthus graecizans* are comparable with those of *Amaranthus viridis* in the same genus reported [35]. The results of *Rumex abyssinicus*, *Opuntia ficus*-*indica*, *Amaranthus graecizans,* and *Urtica simensis* are consistent with the phytochemical study of wild edible plants consumed by rural communities in northern Uganda [35]. The nutritional value of food, colour, taste, smell, antioxidant, anticarcinogenic, antihypertensive, anti-inflammatory, antibacterial, immunity-stimulating, and cholesterol-lowering qualities are determined by these metabolites in addition to their significance for the plant itself [4]. Its use as a therapeutic plant is probably explained by the existence of secondary metabolites [36].

## 4. Conclusions

The results of the current study showed that edible wild plants have the potential to provide humans with all the necessary nutrients. The high amount of protein, carbs, fibre, and fat was discovered in *Amaranthus graecizans, Erucastrum abyssinicum, Urtica simensis, Opuntia ficus-indica*, and *Rumex abyssinicus*. Additionally, these plants have been discovered to be a valuable source of essential nutrients such as calcium, zinc, and iron. These plants are believed to be appropriate for human eating due to their rich nutritional profile, and sufficient safeguards may be put in place to prevent disorders brought on by malnutrition. Following the phytochemical analysis of the extracts of these consumed delicious foods, it was found that substantial phytochemicals were present such as phenols, saponins, alkaloids, flavonoids, tannins, and steroids. Wild edible plants were found to contain a number of chemicals, highlighting their great therapeutic potential. These compounds were found in edible wild plants, demonstrating the significant potential for healing of these plants.

## Figures and Tables

**Table 1:** Proximate composition of five edible wild plants in the study area.

Plant species	Proximate analysis (mg/100 g dry weight)				
	*Amaranthus graecizans* (leaves)	*Erucastrum abyssinicum* (leaves)	*Opuntia ficus-indica* (fruits)	*Rumex abyssinicus* (young shoots)	*Urtica simensis* (leaves)
Moisture	40.8 ± 0.00^de^	29.90 ± 0.00^c^	12.4 ± 0.00^a^	39.53 ± 0.63^de^	25 ± 0.00^b^
Ash	24.7 ± 0.15^e^	4.97 ± 0.17^c^	1.7 ± 0.00^b^	0.9 ± 0.00^a^	21.30 ± 0.00^d^
Fat	8.40 ± 0.00^c^	6.87 ± 0.03^b^	11 ± 0.00^d^	14.07 ± 0.03^e^	4.40 ± 0.6^a^
Fiber	9.70 ± 0.00^b^	7.80 ± 0.2^a^	18.80 ± 0.00^d^	34.70 ± 0.25^e^	10.47 ± 0.07^c^
Protein	14.97 ± 0.03^d^	17.47 ± 0.03^e^	11.70 ± 0.00^b^	9.53 ± 0.03^a^	12.30 ± 0.00^c^
Carbohydrate	1.43 ± 0.14^ab^	32.99 ± 0.00^d^	44.4 ± 0.00^e^	1.27 ± 0.58^ab^	26.53 ± 0.12^c^
Energy (Kcal/100 g)	141.2 ± 0.23^b^	263.46 ± 0.30^c^	326.4 ± 0.00^e^	169.8 ± 0.38^ab^	194.9 ± 0.12^d^

NB: the values are the means of three independent composite sample analyses (on a DW basis) SD. At *p* < 0.05, different superscripts down the column are significantly different.

**Table 2:** Qualitative phytochemical analysis of selected wild edible plants.

Phytochemicals	Qualitative phytochemical analysis				
	*Amaranthus graecizans* (leaves)	*Erucastrum abyssinicum* (leaves)	*Opuntia ficus-indica* (fruits)	*Rumex abyssinicus* (young shoots)	*Urtica simensis* (leaves)
Alkaloids	++	+	+	+	+
Flavonoids	+	+	+	+	+
Phenols	++	+	+	+	+
Triterpenes	+	—	+	+	+
Saponins	+	+	+	+	++
Tannins	+	—	+	+	+

NB: + = low concentration; ++ = high concentration; — = not detected.

## Data Availability

The data used to support the findings of this study are included within the article.
